# Vitamin A deficiency attenuates cardiac rupture in *Stra6-*deficient hearts following ischemic injury

**DOI:** 10.3389/fcvm.2025.1626769

**Published:** 2025-09-16

**Authors:** Yannick Smolenski, Natali Froese, Paolo Galuppo, Christopher Werlein, Anna Gigina, Steven R. Talbot, Sergej Erschow, Dirk Wedekind, Robert Geffers, Norbert B. Ghyselinck, Heike Bähre, Jan C. Kamp, Lavinia Neubert, Melanie Ricke-Hoch, Johann Bauersachs, Christian Riehle

**Affiliations:** ^1^ Department of Cardiology and Angiology, Hannover Medical School, Hannover, Germany; ^2^ Institute of Pathology, Hannover Medical School, Hannover, Germany; ^3^ Institute for Laboratory Animal Science, Hannover Medical School, Hannover, Germany; ^4^ Helmholtz Center for Infection Research, Research Group Genome Analytics, Braunschweig, Germany; ^5^ Institut de Génétique et de Biologie Moléculaire et Cellulaire (IGBMC), Département de Génétique Fonctionnelle et Cancer, Centre National de la Recherche Scientifique (CNRS UMR7104), Institut National de la Santé et de la Recherche Médicale (INSERM U1258), Université de Strasbourg, Illkirch, France; ^6^ Research Core Unit Metabolomics, Institute of Pharmacology, Hannover Medical School, Hannover, Germany; ^7^ Department of Respiratory Medicine, Hannover Medical School, Hannover, Germany; ^8^ German Center for Lung Research (DZL), Biomedical Research in Endstage and Obstructive Lung Disease Hannover (BREATH), Hannover, Germany

**Keywords:** myocardial infarction, heart failure, cardiac remodeling, wnt signaling, vitamin A, stimulated by retinoic acid gene 6

## Abstract

**Background:**

Stimulated by retinoic acid gene 6 (STRA6) is a cell surface receptor that regulates cellular uptake of vitamin A metabolites and cardiac development. We hypothesized that *Stra6* expression attenuates ischemic injury-induced heart failure following myocardial infarction (MI) by vitamin A-dependent mechanisms.

**Methods:**

MI was induced in mice with *Stra6* germline deletion, vitamin A deficiency (VitAD) by combined *lecithin-retinol acyltransferase (Lrat)* germline deletion and feeding with a vitamin A-deficient diet. Contractile function was determined by transthoracic echocardiography, cardiac structure was assessed by histological analysis, and gene profiling was performed by RNA sequencing.

**Results:**

*Stra6* deletion and VitAD did not impact contractile function and cardiac structure under basal conditions. *Stra6* deficiency resulted in myocardial rupture, with the majority of mice dying by 4 days post-MI, which additional VitAD attenuated. Interestingly, contractile function, mRNA expression of heart failure markers, and cardiac structure were not different between groups 3 days post-MI. Gene profiling 3 days post-MI revealed decreased Wnt signaling in *Stra6*-deficient relative to wildtype hearts, which was reversed by VitAD.

**Conclusion:**

The present study identifies an unexpected role for VitAD, which preserves Wnt signaling and attenuates cardiac rupture in *Stra6*-deficient hearts following ischemic injury.

## 1 Introduction

Vitamin A (retinol) is critical for the development and energy homeostasis of mammalian cells, as evidenced by growth retardation and congenital malformations, including cardiac defects, under vitamin A-deficient conditions (1, 2). Retinoids are defined as synthetic and natural derivatives of vitamin A. The two most important pathways for retinoid delivery and cellular uptake comprise physical association with chylomicrons and binding to the adipokine retinol-binding protein 4 (RBP4), the predominant form of retinol delivery (3, 4). Stimulated by retinoic acid gene 6 (STRA6) is a cellular membrane protein and receptor for RBP4, which bidirectionally mediates retinol transport across the cellular membrane (6). Retinol-loaded RBP4 (holo-RBP4) forms a complex with transthyretin (TTR) to prevent glomerular filtration (7). STRA6 binds holo-RBP4 with high affinity and mediates cellular retinol uptake (5). Intracellular retinol can be esterified to retinyl esters, the storage form of retinoids, which is mainly transduced by lecithin-retinol acyltransferase (LRAT) (3, 4). LRAT and cellular retinol binding protein-1 (CRBP1) stimulate cellular retinol uptake (6). In contrast, STRA6-facilitated retinol efflux occurs in the presence of apo-RBP4. Moreover, STRA6 mediates retinol exchange between extracellular RBP4 and intracellular CRBP1 (8).

Retinol is also converted to retinoic acid (RA), which activates a broad transcriptional program by binding to the nuclear receptors retinoic acid receptor (RAR) and retinoid X receptor (RXR), which function as heterodimers that bind to retinoic acid response elements (RARE) located in the promoter regions of target genes (9). Previous studies investigated the impact of vitamin A on cardiac function in the context of various stressors, however; the results have not been consistent (10). RA supplementation attenuates adverse left ventricular (LV) remodeling following ischemic injury and pressure overload in rats (11, 12). Similarly, vitamin A deficiency (VitAD) enhances ischemic injury-induced heart failure (HF) in rats (13). Using a murine model, we recently identified a transcriptional program by which vitamin A preserves cardiac energetic gene expression in diet-induced obesity that might attenuate the subsequent onset of contractile dysfunction (14). In contrast, VitAD attenuates adverse remodeling following ischemic injury in mice (15). These reports highlight the complex aspects of retinoid metabolism in the context of various models and stressors.

*Stra6* is expressed in cardiac tissue and *Stra6* mutations are associated with congenital defects, including microphthalmia/anophthalmia and cardiac malformations (Matthew-Wood syndrome) (5, 16). Previous reports suggest that *Stra6* might be cardioprotective in the context of ischemia/reperfusion (I/R) injury (17–19). However, the impact of *Stra6* expression on ischemic heart disease and its correlation with vitamin A availability remains to be determined. To address this important question, we generated a murine model with combined *Stra6* deletion and VitAD that was subjected to surgically induced myocardial infarction (MI).

## 2 Materials and methods

### 2.1 Animals

*Lrat* germline knockout mice (*Lrat*^−/−^) were purchased from Jackson Laboratories (strain #018866) (20). *Lrat*^−/−^ mice were cross-bred with *Stra6* germline knockout mice (*Stra6*^−/−^) to generate mice with germline deletion of both *Lrat* and *Stra6* (*Lrat*^−/−^ x *Stra6*^−/−^; DKO) (21). Mice were on a pure C57/Bl6J genetic background, and genotyping was performed as previously described (20, 21). Wildtype (WT) and *Stra6*^−/−^ mice were fed a standard chow diet (C1000, Altromin, Lage, Germany). *Lrat*^−/−^ and DKO mice received a vitamin A-deficient diet (C1016, Altromin, Lage, Germany). Dietary treatment was initiated at 4–6 weeks of age and continued until tissue harvest. MI was induced after 4 weeks of dietary treatment at the age of 8–10 weeks. Animals were housed in a 14 h light/10 h dark cycle with *ad libitum* access to food and water. Studies were performed in male mice. For tissue harvest, mice were euthanized by cervical dislocation under isoflurane anesthesia. All experiments were performed in accordance with the ARRIVE guidelines and with protocols approved by local state authorities (Niedersächsisches Landesamt für Verbraucherschutz und Lebensmittelsicherheit; protocol numbers: 23/00360 and 24/00719), which conform to the *Guide for the Care and Use of Laboratory Animals* published by the US National Institutes of Health.

### 2.2 Quantitative RT-PCR analysis

Total RNA from the infarct border zone was isolated using the NucleoSpin RNA kit (Macherey-Nagel, Düren, Germany) and cDNA synthesis was performed (MaximaTM H Minus First Strand cDNA Synthesis kit, Thermo Fisher Scientific, Waltham, MA, USA) according to the manufacturers’ instructions. Quantitative RT-PCR analysis was performed using the Luna® Universal qPCR Master Mix (New England Biolabs, Ipswich, MA, USA) and a LightCycler® 96 PCR system (Roche, Mannheim, Germany) (22). Primers are listed in Supplementary Table S1.

### 2.3 Measurement of retinoid levels

Retinoids were extracted from liver tissue. All-*trans*-retinal [lower limit of quantification (LLOQ): 0.992 pmol/sample] and all-*trans*-retinol levels (LLOQ: 3.4 pmol/sample) were determined as previously described (14).

### 2.4 Coronary artery ligation

MI was induced by left anterior descending coronary artery (LAD) ligation. Mice were pre-treated with metamizole (1A Pharma, Holzkirchen, Germany) dissolved in drinking water (500 mg/kg) the day before surgery. Before surgery, mice were treated by intraperitoneal injection of butorphanol (2 mg/kg; Cp-Pharma, Burgdorf, Germany) and subcutaneous injection of carprofen (5 mg/kg; Cp-Pharma, Burgdorf, Germany). Anesthesia was induced with 2%–4% isoflurane dissolved in oxygen. After oral intubation, mice were mechanically ventilated (MiniVent Type 683, Harvard Apparatus, Holliston, MA, USA), and anesthesia was maintained with 1%–4% isoflurane dissolved in oxygen. Mice were placed on a heating pad (37°C), and an eye care solution was applied to prevent corneal injury. Local anesthesia (a combination of 0.5% lidocaine and 0.25% bupivacaine) was applied before a left thoracotomy in the fifth intercostal space. After opening the pericardium, the LAD was ligated using a 6-0 Prolene suture (Ethicon, Norderstedt, Germany). MI was evident from LV discoloration. The thorax was closed, and mice recovered at 32°C. Animals received subcutaneous injections of carprofen (5 mg/kg) for an additional 3 days post-surgery. Sham-operated mice were subjected to similar surgery, except that no ligature around the LAD was placed.

### 2.5 Transthoracic echocardiography

Transthoracic echocardiography was performed under isoflurane anesthesia (induced at a concentration of 5% and sustained with 1% isoflurane). Mice were placed in the supine position on a heating pad (37°C). An eye care solution was applied to prevent corneal injury. Two-dimensional B-mode images were acquired, and endocardial silhouettes were traced manually. Ejection fraction was determined in long-axis projection using the VevoStrain software (VisualSonics Inc.). LV end-diastolic area (LVEDA) and LV end-systolic area (LVESA) were determined in long-axis parasternal projections. Fractional area change (FAC) was calculated as [(LVEDA—LVESA)/LVEDA] * 100 (14, 22).

### 2.6 Histological analysis and quantification of cardiac fibrosis

Paraffin sections of cardiac tissue were prepared, stained with hematoxylin and eosin (H&E) or Picrosirius red (PSR) staining solutions, and quantified as previously described (14). As previously reported, hearts were embedded in OCT, cut into 10-µm-thick sections, and stained with PSR staining solution for transverse sections (23).

### 2.7 Immunoblotting analysis

Protein extraction from the infarct border zone, immunoblotting, and densitometric analysis were performed as previously described (22). Antibodies used for immunoblotting are listed in Supplementary Table S2.

### 2.8 RNA sequencing

Total RNA from the infarct border zone was isolated using the NucleoSpin RNA kit (Macherey-Nagel, Düren, Germany). Quality/integrity control of total RNA was performed using a 2100 Bioanalyzer System (Agilent Technologies, Waldbronn, Germany) (22). The RNA sequencing library was generated from 500 ng of total RNA using the NEBNext® Ultra^TM^ II Directional RNA Library Prep Kit for Illumina® (New England BioLabs, Frankfurt, Germany) with the NEBNext® Poly(A) mRNA Magnetic Isolation Module according to the manufacturer's instructions. RNA libraries were sequenced using a NovaSeq 6000 system and the NovaSeq 6000 S1 Reagent Kit (100 cycles, paired end run) with an average of 3 * 10^7^ reads per sample (Illumina, San Diego, CA, USA). Quality reports were generated for each FASTQ file using the FASTQC tool (https://www.bioinformatics.babraham.ac.uk/projects/fastqc). Raw FASTQ files were trimmed on base call quality and sequencing adapter contamination using fastq-mcf (https://expressionanalysis.github.io/ea-utils/). Reads shorter than 15 bp were removed from FASTQ files. Next, trimmed reads were aligned to the murine reference genome using the open-source short-read aligner STAR with settings according to the log file (24). Data analysis was performed with the statistical programming language R (v4.1.1) (25). Feature counts were determined using the R package “Rsubread” (v2.2.6), and transcript annotation was performed using the R package “bioMaRt” (v2.44.4) (26, 27). The R package “DESeq2” (v1.32.0) was used to evaluate differential gene expression (28–31). To ensure robust expression analysis, only transcripts with a count of at least 10 in about 40% of total samples were used for further processing. A total of two libraries were removed from further analysis based on outlier properties in principal component analysis (PCA) and Cook's distance to assess sample-level variance. Transcripts involved in retinoid signaling and metabolism were identified based on previous publications and their enrichment in the top 500 genes contributing to PC2 was determined by Fisher's Exact Test (32–40). The R package clusterProfiler was applied to the top 500 genes contributing to PC2 and gene ontology (GO) term enrichment analysis was performed. The identified GO terms were filtered to extract GO terms containing keywords related to retinoid biology, which identified the following pathways: “retinoic acid metabolic process”, “retinoic acid biosynthetic process”, and “retinoid metabolic process”. Functional analysis was performed using the Ingenuity Pathway Analysis (IPA) tool (Qiagen, Germantown, MD, USA; https://digitalinsights.qiagen.com/products-overview/discovery-insights-portfolio/analysis-and-visualization/qiagen-ipa). Transcripts were classified based on KEGG pathways [Wnt signaling pathway: mmu04310, cytoskeleton in muscle cells: mmu04820, extracellular matrix (ECM)-receptor interaction: mmu04512; https://www.genome.jp/kegg]. Gene set enrichment analysis (GSEA) was performed with software version 4.1.0 (41–44). Cumulative distribution analysis was performed with R. *P*-values are reported for two-sided Kolmogorov–Smirnov tests (cutoff for significance: *p* < 0.05). Spline interpolation was used for cumulative distribution function (ECDF) plots for visual clarity.

### 2.9 Statistics

Data are expressed as mean ± SEM. Two-way ANOVA was performed to analyze differences after *Stra6*^−/−^ by *Lrat*^−/−^ in non-operated groups, after *Stra6*^−/−^ by VitAD in Sham-operated groups at time points investigated, and after *Stra6*^−/−^ by VitAD in MI-operated groups at the 3-day time point, each followed by Holm-Šídák *post hoc* analysis. T-Tests were performed to analyze differences after MI relative to Sham, which had the same *Stra6* expression and vitamin A availability. One-way ANOVA was performed to analyze differences between MI-operated groups at the 2-week and 4-week time points, followed by Holm-Šídák *post hoc* analysis. Survival was analyzed using a log-rank test and adjusted for multiple testing using the Bonferroni method. Statistical analyses were performed using GraphPad Prism software version 8.0 (GraphPad Software, San Diego, CA, USA) and for RNA sequencing analysis as described above. For all analyses, a *p*-value of <0.05 was considered significantly different.

## 3 Results

### 3.1 Induction of VitAD independent of *Stra6* expression

**Figure 1:**
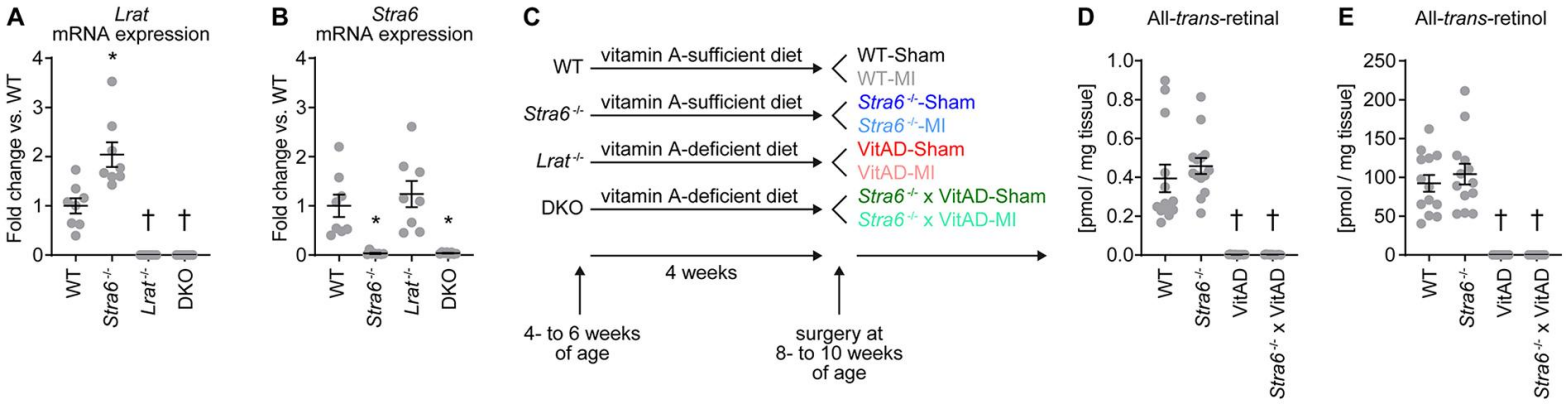
Experimental setup and induction of vitamin A deficiency. **(A,B)** *Lrat* (#, $, &) and *Stra6* (#) mRNA expression normalized to *Gapdh* in left ventricular tissue from mice with germline deletion of *Stra6* (*Stra6*^−/−^), *Lrat* (*Lrat*^−/−^) or both (DKO) presented as fold change vs. wildtype (WT); *n* = 8 (* *p* < 0.05 vs. *Stra6*^+/+^ same *Lrat* allele expression, † *p* < 0.05 vs. *Lrat*^+/+^ same *Stra6* allele expression). Two-way ANOVA was performed to analyze differences by *Stra6*^−/−^ and *Lrat*^−/−^ (# *p* < 0.05 for *Stra6*^−/−^, $ *p* < 0.05 for *Lrat*^−/−^, and & *p* < 0.05 for the interaction between *Stra6*^−/−^ and *Lrat*^−/−^). **(C)** An experimental setup was used to treat *Lrat*^−/−^ and DKO mice with a vitamin A-deficient diet to induce vitamin A deficiency (VitAD). Dietary treatment was started at 4–6 weeks of age and was continued until tissue harvest. Ischemic injury by myocardial infarction (MI) was induced after 4 weeks of dietary treatment at the age of 8–10 weeks. **(D)** All-*trans*-retinal and **(E)** all-*trans*-retinol levels in liver tissue 3 days post-surgery ($ each); *n* = 12–14. Data are pooled from Sham- and MI-operated mice with the same genotype and vitamin A availability († *p* < 0.05 vs. vitamin A sufficiency same *Stra6* allele expression). Two-way ANOVA was performed to analyze differences after *Stra6*^−/−^ by VitAD ($ *p* < 0.05 for VitAD).

RT-PCR analysis of LV tissue confirmed deletion of *Lrat* in *Lrat*^−/−^ and DKO mice, *Stra6* in *Stra6*^−/−^ and DKO mice, and both in DKO mice (Figures 1A,B). Liver retinoid levels reflect whole body vitamin A status (45, 46). *Lrat* germline deletion (*Lrat*^−/−^) decreases hepatic retinoid levels and impairs tissue retinoid levels in the absence of dietary vitamin A (15, 20, 47, 48). To test the hypothesis that *Stra6* expression attenuates ischemic injury-induced HF following MI by vitamin A-dependent mechanisms, we subjected *Lrat*^−/−^ and DKO mice to a vitamin A-deficient diet starting at 4- to 6 weeks of age (groups indicated as “VitAD” and “*Stra6*^−/−^ × VitAD” respectively). WT and *Stra6*^−/−^-mice were fed with a vitamin A-sufficient diet (groups indicated as “WT” and “*Stra6*^−/−^” respectively). MI was induced after 4 weeks of dietary treatment and was continued until tissue harvest. Sham-operated mice with the same genotype and dietary treatment served as controls (Figure 1C). All-*trans*-retinal and all-*trans*-retinol levels were nearly absent in livers from the VitAD and *Stra6*^−/−^ × VitAD groups (all-*trans*-retinal [pmol/mg tissue]: WT 0.394 ± 0.071, *Stra6*^−/−^ 0.458 ± 0.041, VitAD 0.002 ± 0.000, *Stra6*^−/−^ × VitAD 0.002 ± 0.000; all-*trans*-retinol [pmol/mg tissue]: WT 92.3 ± 10.8, *Stra6*^−/−^ 104.1 ± 13.3, VitAD not detectable, *Stra6*^−/−^ × VitAD not detectable; Figures 1D,E). These data confirm VitAD independent of *Stra6* expression. Tissue RA levels are regulated by CYP26 hydroxylases, including CYP26A1. RA mediate CPY260 enzyme expression in a feedback regulation loop and CPY26 levels correlate with vitamin A availability (49–51). *Cyp26a1* mRNA expression trended to decrease in the VitAD-Sham relative to the WT-Sham group (−61.5%, *p* = 0.059; Supplementary Figure S1), which suggests decreased cardiac RA levels in the VitAD group.

### 3.2 VitAD attenuates increased mortality in *Stra6^−/−^* mice following ischemic injury independent of STAT3 and STAT5/Akt signaling

**Figure 2:**
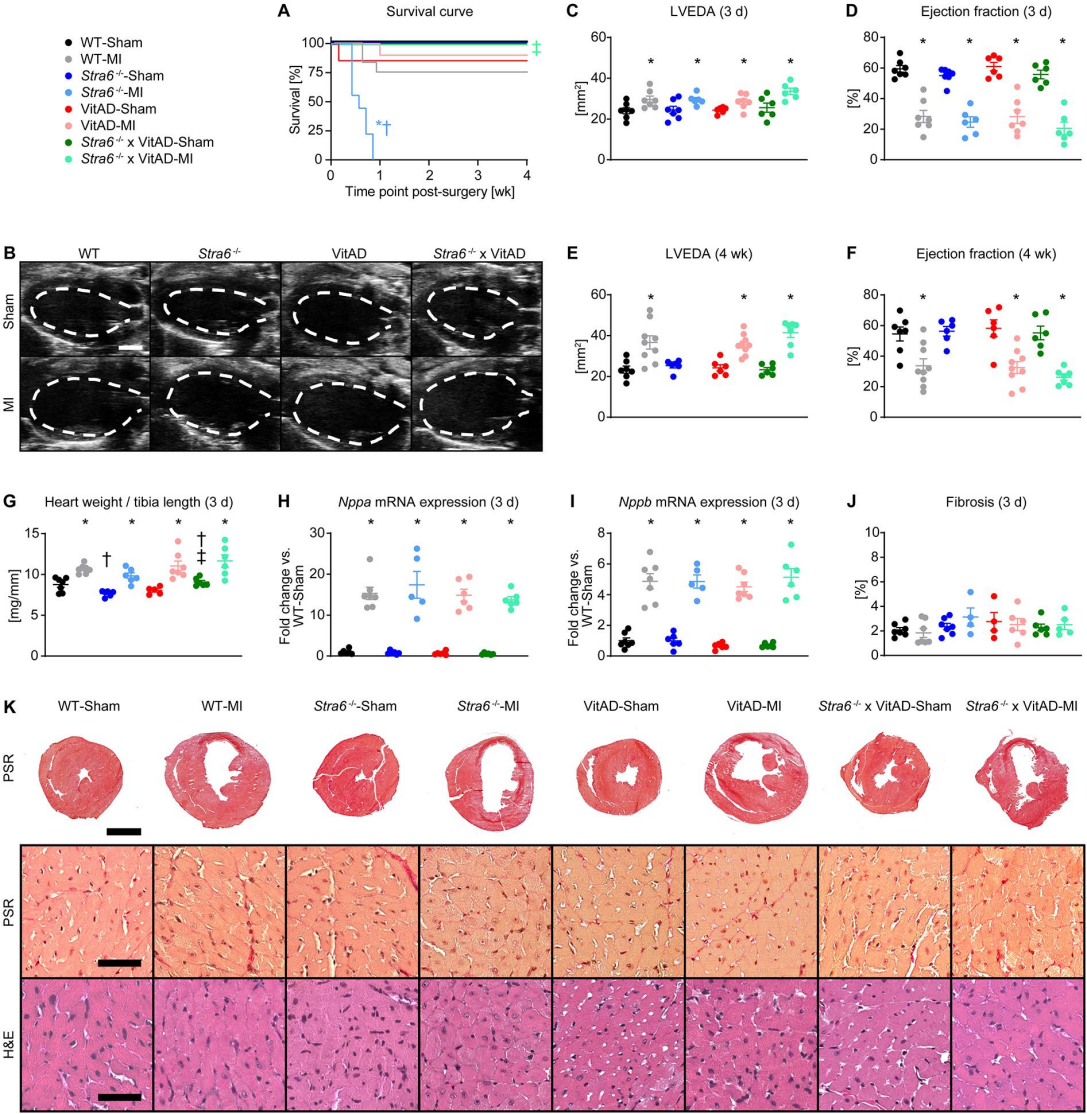
Vitamin A deficiency attenuates increased mortality in Stra6^−/−^ mice following ischemic injury. **(A)** Survival curve (*n* = 6–12). **(B)** Representative B-mode echocardiography images at end-diastole in long axis projection 3 days post-surgery from mice as indicated. The dashed line indicates left ventricular end-diastolic area (LVEDA), scale bars: 2 mm. **(C,D)** LVEDA and ejection fraction 3 days post-surgery (*n* = 6–7). **(E,F)** LVEDA and ejection fraction 4 weeks post-surgery (*n* = 6–9). **(G)** Heart weights normalized to tibia length 3 days post-surgery (*n* = 5–7; &, $$). **(H,I)** mRNA expression of heart failure markers **(H)** *Nppa* and **(I)** *Nppb* ($) in the infarct border zone 3 days post-surgery, each normalized to *Gapdh* and presented as fold change vs. WT-Sham (*n* = 5–7). **(J)** Quantification of fibrotic area of transverse heart sections 3 days post-surgery (*n* = 4–7). **(K)** Representative transverse heart sections stained with Picrosirius red (PSR; scale bars: 2 mm) and representative sections of the infarct border zone stained with PSR (scale bars: 50 µm) and hematoxylin and eosin (H&E; scale bars: 50 µm) 3 days post-surgery. Data are reported as mean values ± SEM. * *p* < 0.05 vs. Sham same *Stra6* expression and vitamin A availability, † *p* < 0.05 vs. *Stra6*^+/+^ same surgery and vitamin A availability, ‡ *p* < 0.05 vs. vitamin A sufficiency same surgery and *Stra6* expression. Two-way ANOVA was performed to analyze differences between Sham-operated groups by *Stra6*^−/−^ and VitAD ($ *p* < 0.05 for VitAD, and & *p* < 0.05 for the interaction between *Stra6*^−/−^ and VitAD). Two-way ANOVA was performed to analyze differences between MI-operated groups by *Stra6*^−/−^ and VitAD at the 3-day time point ($$ *p* < 0.05 for VitAD).

Following ischemic injury, we observed increased mortality in *Stra6^−/−^* mice, with the majority of mice dying by 4 days post-MI. Mortality was not increased in the other groups investigated relative to Sham-operated mice (Figure 2A). Autopsies revealed cardiac rupture in all *Stra6^−/−^* mice post-MI as diagnosed by the presence of a blood clot in the chest cavity and around the heart. Interestingly, transthoracic echocardiography revealed HF independent of vitamin A status and *Stra6* expression as indicated by a similar increase in LVEDA and a similar decrease in ejection fraction 3 days post-MI (Figures 2B–D; Supplementary Table S3). LVEDA similarly increased, and ejection fraction similarly decreased in MI-operated groups 4 weeks post-surgery (Figures 2E,F; Supplementary Table S3). Heart weights normalized to tibia length (Figure 2G; Supplementary Table S4), and mRNA expression of HF markers (Figures 2H,I) increased 3 days post-MI relative to Sham-operated groups independent of vitamin A status and *Stra6* expression. Similarly, HF marker mRNA expression was increased 4 weeks post-MI relative to Sham-operated groups independent vitamin A status and *Stra6* expression (Supplementary Figure S2). Quantification of fibrotic tissue showed no difference between groups at the 3-day time point (Figures 2J,K). The non-receptor tyrosine kinase janus kinase 2 (JAK2) is recruited to STRA6, which activates signal transducer and activator of transcription 5 (STAT5) (18). I/R activates STAT5A, and *Stat5a*-deficient hearts cannot be preconditioned (17). STAT5 activates the PI-3 kinase (PI3K)/Akt signaling cascade, which is cardioprotective under conditions of ischemic preconditioning (IPC) (17, 19). Similarly, STRA6 activates the JAK2/STAT3 signaling module and STAT3-mediated signaling is cardioprotective following I/R (52, 53). We hypothesized that *Stra6* expression is cardioprotective following ischemic injury by preserving STAT3 and STAT5 signaling. Interestingly, STAT3, but not STAT5/Akt signaling was activated following ischemic injury independent of *Stra6* expression and vitamin A availability 3 days post-surgery (Supplementary Figure S3). Together, these data reveal that *Stra6* deletion does not impact cardiac structure and contractile function under basal conditions. Moreover, VitAD attenuates increased mortality in *Stra6^−/−^* mice following ischemic injury independent of STAT3 and STAT5/Akt signaling.

### 3.3 VitAD reverses the decrease in Wnt signaling in *Stra6*-deficient hearts following ischemic injury

**Figure 3:**
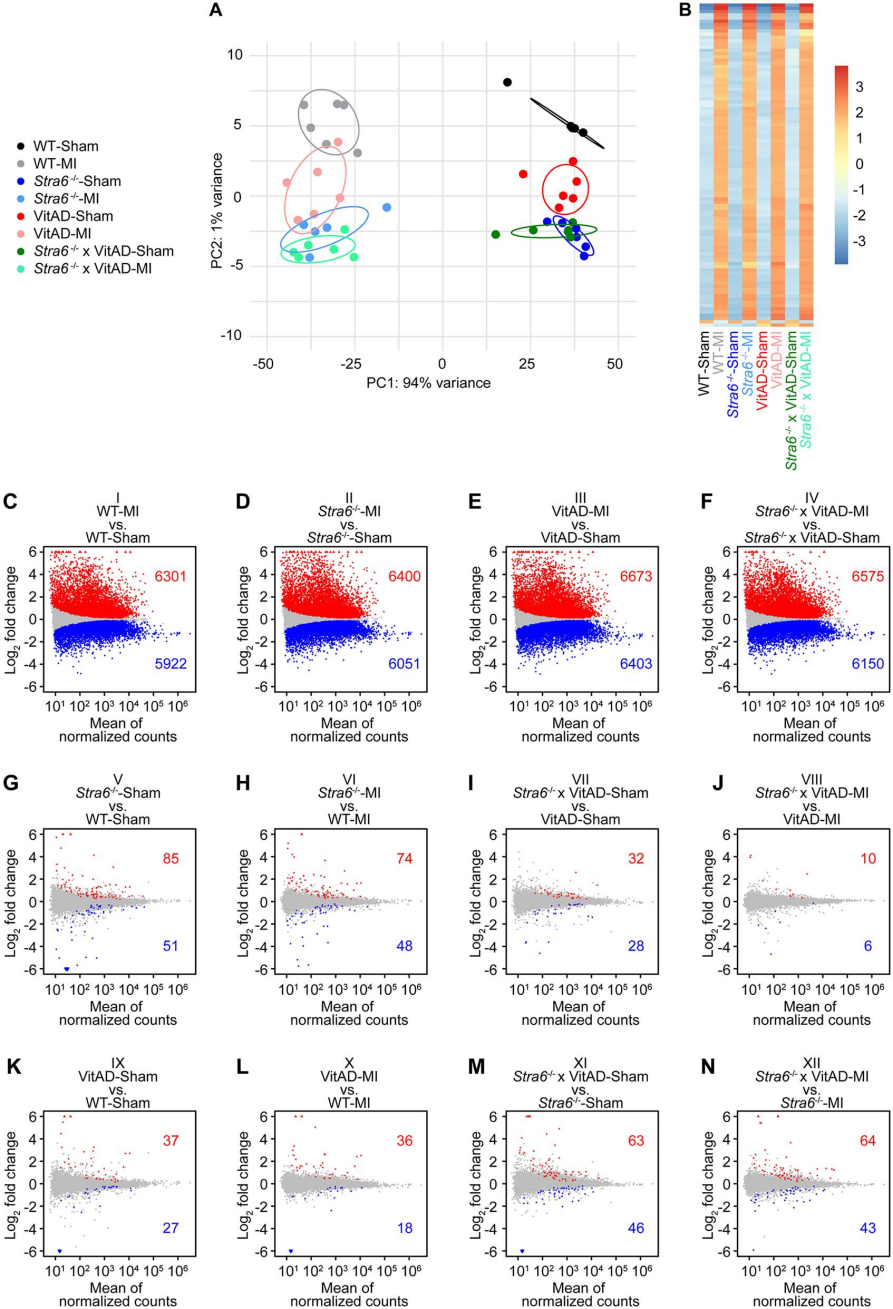
Gene expression 3 days post-surgery as determined by RNA sequencing. **(A)** Principal component analysis to visualize global gene expression clusters by surgery, *Stra6* expression, and vitamin A availability (*n* = 5–6). **(B)** Heatmap of RNA sequencing count data corresponding to the 100 genes with the greatest variance across samples. Data are clustered by row after applying the regularized log transformation function in DESeq2. **(C–N)** Gene expression presented as MA plots showing log_2_ fold change vs. mean of normalized counts for comparisons as indicated. Red and blue dots indicate differentially up- or downregulated genes, and gray dots indicate not differentially regulated genes (cutoff: FDR < 0.01; *n* = 5–6). Red triangles indicate genes with a log_2_ fold change >6, and blue triangles indicate genes with a log_2_ fold change <−6.

To delineate the mechanisms contributing to cardiac rupture in *Stra6-*deficient heart post-MI and the reversal under VitAD conditions, we performed differential gene expression analysis by RNA sequencing using tissue from the infarct border zone. A total of 19,023 transcripts were considered for analysis (Supplementary Table S5). Principal component analysis (PCA) revealed a predominant effect of surgery performed on gene expression (Figure 3A), which is supported by a heatmap presenting the top 100 genes with the greatest variance across groups (Figure 3B). Using a list of previously identified genes that play a key role in retinoid signaling and metabolism (Supplementary Table S6), we identified that retinoid-related transcripts are significantly enriched in the top 500 genes contributing to PC2 (*p* = 0.0338, odds ratio = 11.91; Supplementary Table S7; Supplementary Figure S4A), but not in the top 500 PC1-contributing genes. Gene ontology enrichment analysis using PC2 loading genes identified significantly enriched terms related to retinoid biology, including “retinoic acid metabolic process”, “retinoic acid biosynthetic process”, and “retinoid metabolic process” (Supplementary Figure S4B). These results suggest that PC2 captures transcriptional variation related to retinoid acids.

We explored differences in gene expression using pairwise comparisons of groups. We investigated 12 comparisons representing differences in surgery performed (comparisons I–IV), in *Stra6* expression (comparisons V–VIII), and vitamin A availability (comparisons IX to XII; Figures 3C–N). A total of 6,301 transcripts was induced and 5,922 were repressed in WT-MI vs. WT-Sham (comparison I), 6,400 induced/6,051 decreased in *Stra6*^−/−^-MI vs. *Stra6*^−/−^-Sham (comparison II), 6,673 induced/6,403 decreased in VitAD-MI vs. VitAD-Sham (comparison III), and 6,575 induced/6,150 decreased in *Stra6*^−/−^ × VitAD-MI vs. *Stra6*^−/−^ × VitAD-Sham (comparison IV, Figures 3C–F, cutoff: FDR < 0.01). In contrast, the expression of a relatively small number of genes was altered for comparisons V–XII after applying the same cutoff (Figures 3G–N). These data support our PCA and indicate a predominant effect of surgery performed relative to *Stra6* expression and vitamin A availability on gene expression. Moreover, ischemic injury mediates the expression of retinoid signaling and metabolism genes (Supplementary Figure S5; Supplementary Table S6). Cumulative distribution analysis showed decreased expression of cytoskeleton and ECM-receptor interaction genes in Sham-operated groups following *Stra6* deletion and vitamin A deficiency, which was reversed by the combination of both (Supplementary Figure S6).

**Figure 4:**
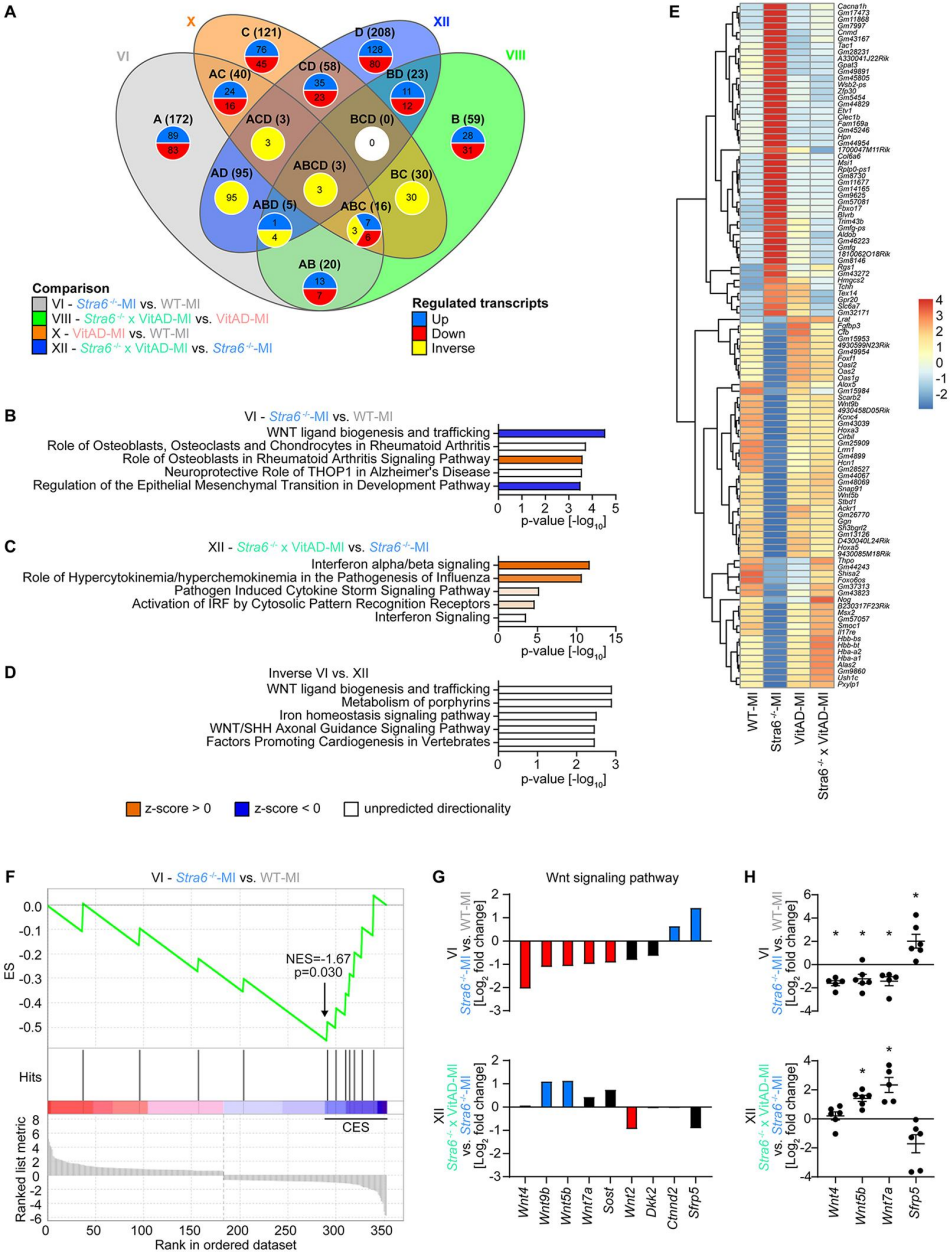
Differential gene expression analysis 3 days post-ischemic injury as determined by RNA sequencing. **(A)** Venn diagram illustrating the number of altered transcripts in myocardial infarction (MI)-operated groups for comparisons as indicated (cutoff: |log_2_ fold change| > 0.6 and *p* < 0.05). **(B–D)** Top canonical pathways identified by Ingenuity Pathway Analysis (IPA) for comparisons as indicated (cutoff: |log_2_ fold change| > 0.6 and *p* < 0.05). **(E)** Heatmap of RNA sequencing count data representing regulated transcripts for comparison VI (*Stra6*^−/−^-MI vs. WT-MI) relative to comparison XII (*Stra6*^−/−^ × VitAD-MI vs. *Stra6*^−/−^-MI) in the opposite direction (cutoff: |log_2_ fold change| > 0.6 and *p* < 0.05; *n* = 5–6). Data are clustered by row after applying the regularized log transformation function in DESeq2. **(F)** Enrichment plot for Wnt signaling genes by gene set enrichment analysis (GSEA) for comparison VI (*Stra6*^−/−^-MI vs. WT-MI) 3 days post-surgery. The *x*-axis indicates Wnt signaling genes represented in the gene set (indicated as “hits”), and the *y*-axis represents enrichment scores (ES). The green line depicts the enrichment profile and connects ES and genes; NES, normalized enrichment score. The arrow indicates the point of maximal distance of ES from the baseline as determined by genes of the core enrichment set (CES). Lower plots in gray present all genes in rank order according to the signal-to-noise metric for comparison VI (cutoff: |log_2_ fold change| > 0.6 and *p* < 0.05). The dashed line separates Wnt signaling pathway genes that are positively (red) and negatively (blue) correlated with loss of *Stra6* expression following ischemic injury, with the colored band indicating the degree of correlation. **(G)** Expression of Wnt signaling pathway transcripts presented as mean values for comparisons as indicated (cutoff: |log_2_ fold change| > 0.6 for comparison VI or comparison XII; *n* = 5–6). Red and blue bars indicate down and upregulated, respectively. Black bars are not regulated (cutoff: *p* < 0.05). **(H)** Expression of Wnt signaling pathway transcripts as determined by RT-PCR analysis (*n* = 5–7; * *p* < 0.05).

Next, we focused on differentially expressed genes between MI-operated groups (comparisons VI, VIII, X, and XII; Figure 4A; Supplementary Table S8). We investigated comparison VI (*Stra6*^−/−^-MI vs. WT-MI) to identify transcripts by which *Stra6* deletion results in cardiac rupture post-MI and comparison XII (*Stra6*^−/−^ × VitAD-MI vs. *Stra6*^−/−^-MI) to delineate transcripts by which VitAD attenuates this effect. Interestingly, IPA identified decreased wingless/int-1 protein (Wnt) ligand biogenesis and trafficking as the top canonical pathway for comparison VI and increased interferon alpha/beta signaling for comparison XII (Figures 4B,C; cutoff: |log_2_ fold change| > 0.6 and *p* < 0.05). We next investigated transcripts that were altered in comparisons VI and XII in the opposite direction (gene sets indicated as ABCD, ABD, ACD, and AD in Figure 4A) and identified a total of 105 transcripts (Figures 4D,E; Supplementary Table S8; cutoff: |log_2_ fold change| > 0.6 and *p* < 0.05). Of note, Wnt ligand biogenesis and trafficking was also the top canonical pathway for the identified 105 inversely regulated transcripts (Figure 4D). Moreover, GSEA identified decreased expression of Wnt signaling genes for comparison VI (cutoff: |log_2_ fold change| > 0.6 and *p* < 0.05; Figure 4F), which supports the results of the IPA performed (Figure 4B). The oppositely expressed Wnt signaling transcripts between comparisons VI and XII comprise *Wnt5b, Wnt9b,* and *secreted frizzled-related protein (Sfrp5)*, which have been associated with the pathogenesis of HF (Figure 4G) (54–58). Results of the RNA sequencing experiment were confirmed by RT-PCR analysis (Figure 4H). Together, these data suggest that VitAD attenuates cardiac rupture in *Stra6*-deficient hearts following ischemic injury, at least in part, by the opposite expression of *Wnt5b, Wnt9b,* and *Sfrp5* and reversing the decrease in Wnt signaling.

## 4 Discussion

The impact of *Stra6* on the heart and ischemic heart disease is incompletely understood. The present study suggests that VitAD preserves Wnt signaling and prevents myocardial rupture in *Stra6-*deficient hearts following ischemic injury.

*Stra6* mediates various signaling pathways, including the Wnt pathway. Wnt signaling regulates embryonic development, including the heart, cell fate determination, and the cardiac response to ischemic injury (56, 57). *Stra6* is an oncogene in gastric tumorigenesis by activating Wnt/ß-catenin signaling (59). Additionally, STRA6 expression is upregulated in murine C57MG mammary epithelial cells following combined stimulation with Wnt-1 and RA (60). Wnt signaling is categorized into the canonical (β-catenin-dependent) and the non-canonical (β-catenin-independent) pathways (57, 61). Canonical Wnt signaling is activated by binding Wnt ligands to a heterodimeric receptor complex consisting of frizzled (FZD) and lipoprotein receptor-related protein (LRP) 5 and 6. Activation status of canonical Wnt signaling is mediated by cytosolic β-catenin levels controlled by a destruction complex, ultimately leading to its degradation. Binding of Wnt ligands to the G protein-coupled receptor FZD and LRP5/6 co-receptors sequesters the destruction complex's components, which increases β-catenin levels. β-catenin mediates transcription by binding to members of the T cell factor (TCF)/lymphoid enhancer factor (LEF) transcription factor family (57, 61). The non-canonical Wnt pathways include the Wnt/planar cell polarity (PCP) pathway, which is critical for development, and the Wnt/Ca^2+^ pathway that regulates numerous pathways, including calmodulin-dependent kinase II (CaMKII), calcineurin, and protein kinase C (PKC) signaling (56, 61). Previous studies reported beneficial effects of Wnt signaling inhibition following ischemic injury and in ischemic heart disease; however, the results have not been consistent (56). For example, inhibition of glycogen synthase kinase-3β (GSK3β), which is part of the β-catenin destruction complex, has been reported to be cardioprotective following ischemic injury. Of note, inhibition of GSK3β is considered to activate Wnt signaling based on decreased GSK3β-targeted β-catenin degradation; however, GSK3β is also involved in numerous additional pathways regulating cardiac remodeling (56).

The present study identified decreased *Wnt5b* and *Wnt9b* expression in *Stra6-*deficient hearts relative to WT hearts post-MI, which the additional VitAD reversed. *Wnt5b* and *Wnt9b* mediate canonical and non-canonical Wnt signaling (58). Therefore, our study suggests an adverse role for decreased *Wnt5b* and *Wnt9b* expression and decreased Wnt signaling following ischemic injury in *Stra6-*deficient hearts. We detected increased *Sfrp5* expression in *Stra6-*deficient hearts relative to WT controls post-MI, which additional VitAD reversed. Importantly, *Sfrp5* is an extracellular inhibitor of the non-canonical Wnt pathway (54, 55). Thus, increased *Sfrp5* expression might further decrease Wnt signaling in *Stra6-*deficient hearts under vitamin A-sufficient conditions, which additional VitAD attenuates. Previous studies using various models indicate that RA-mediated signaling inhibits Wnt signaling. For example, treatment of the transgenic mouse mammary tumor virus (MMTV)-Wnt1 breast cancer model with the RAR*α* agonist Am580 inhibits the Wnt pathway and increases tumor-free survival (62). Similarly, RA repress Wnt signaling, which is required for proper endocrine cell differentiation (63). Moreover, RA inhibits the canonical Wnt pathway in embryonic stem cells while activating non-canonical Wnt signaling (64). Together, these reports are in concert with the present study, which identifies that VitAD activates Wnt signaling following ischemic injury in the context of *Stra6* deletion. The underlying mechanisms by which *Stra6* deletion and additional VitAD mediate Wnt signaling post-MI and in ischemic heart disease require further investigation.

STRA6 mutations cause Matthew-Wood syndrome, which is characterized by malformations, including congenital heart defects (16). The present study reports increased *Lrat* mRNA expression in LV tissue from *Stra6*^−/−^ mice compared to WT under non-stressed conditions, while no difference in *Cyp26a1* mRNA expression in *Stra6*^−/−^-Sham relative to WT-Sham was detected. These data suggest no difference in cardiac retinoid content following *Stra6* deletion post-Sham surgery, which is supported by previous studies (21). STRA6 is the RBP4 membrane receptor and circulating RBP4 levels are associated with cardiovascular disease. Circulating RBP4 levels have been determined in MI patients; however, the results have not been consistent (65–68). RBP4 mRNA and proteins levels are increased in the infarct border zone following MI in mice and in ischemia/hypoxia treated cardiomyocytes. Notably, knockdown of RBP4 in cardiac tissue decreases infarct size and attenuates ischemic injury-induced HF in mice (69). Given the adverse contribution of RBP4 to the response of ischemic injury in mice and the phenotype observed in patients with Matthew-Wood syndrome, RBP4 rather than STRA6 might be a potential therapeutic approach for the treatment of MI.

Limitations of the present study include investigating adult male mice at a relatively young age. This contrasts with patients, who typically suffer from MI and ischemic heart disease at a higher age. Also, sex-related differences in cardiac function post-MI have been reported for murine models, with male mice exhibiting impaired contractile function during decompensation to HF relative to female mice (70, 71). Importantly, the majority of *Stra6*-deficient mice died by 4 days post-MI. Molecular analyses, including differential gene expression analysis, were performed 3 days post-MI. At the same time, the response to ischemic injury is similar in female and male mice during the phase of early compensation 2 weeks post-MI (71). Gene profiling was performed in the infarct border zone, which consists of different cell types. Therefore, the gene profiling experiment cannot discern the impact of *Stra6* deficiency and VitAD on the gene expression of specific cell types in the infarct border zone. VitAD was induced by feeding *Lrat*^−/−^ mice with vitamin A-deficient diet for the duration of 4 weeks, which dramatically decreases serum retinol levels (47). Even though not directly proven, cardiac retinoid supply is therefore likely decreased in the VitAD groups. Retinoid levels were detected in liver tissue, indicative of whole-body vitamin A status (45, 46). Despite our mass spectrometry analysis only detected all-*trans*-retinal and all-*trans*-retinol levels in liver tissue, these data indicate impaired vitamin A availability in the VitAD and VitAD x *Stra6*^−/−^ groups independent of *Stra6* expression. Studies have been performed in mice with germline deletion of *Lrat, Stra6* or both. Therefore, the genetic manipulation in the models used may also impact the immune system and the neuroendocrine axis, which might contribute to the phenotype observed (72, 73).

**Figure 5:**
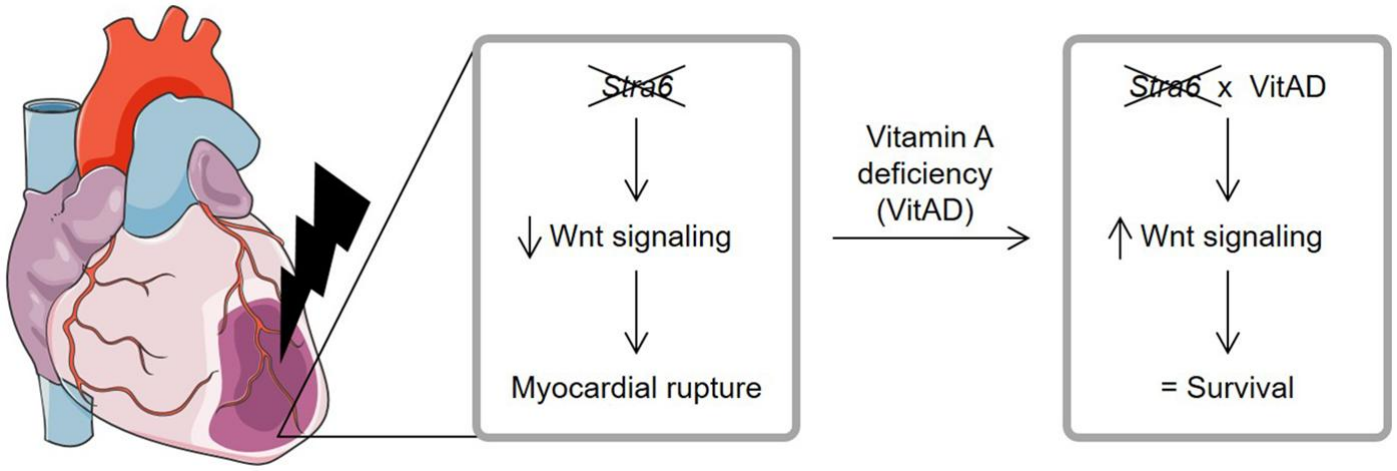
Relationship between Stra6 expression and vitamin A deficiency following ischemic heart injury. *Stra6* deletion decreases Wnt signaling following myocardial infarction and results in myocardial rupture. Additional vitamin A deficiency (VitAD) preserves Wnt signaling in *Stra6*-deficient hearts, prevents myocardial rupture, and prolongs survival. Created using Servier Medical Art, licensed under CC BY 3.0.

In summary, present study identifies an unexpected role for VitAD, which preserves Wnt signaling and attenuates cardiac rupture in *Stra6*-deficient hearts following ischemic injury (Figure 5). These data also extend our knowledge of the complex aspects of *Stra6*-mediated signaling and vitamin A metabolism in the context of ischemic heart disease and emphasize the need for further studies before using vitamin A metabolites to treat cardiovascular disease.

## Data Availability

The original contributions presented in the study are publicly available. This data can be found here: GEO (https://www.ncbi.nlm.nih.gov/geo/), accession number: GSE307187.
