# Supplementary material for: Vitamin A deficiency attenuates cardiac rupture in Stra6-deficient hearts following ischemic injury
Source: Front Cardiovasc Med. 2025 Sep 16;12:1626769. doi: 10.3389/fcvm.2025.1626769 (PMC12479519; doi:10.3389/fcvm.2025.1626769)
Supplement: Supplementary file 1 [file Datasheet1.pdf]

SUPPLEMENTARY MATERIAL

FOR

Vitamin A deficiency attenuates cardiac rupture in *Stra6*-deficient hearts following ischemic injury

Yannick Smolenski[1, †], Natali Froese[1, †], Paolo Galuppo[1], Christopher Werlein[2], Anna Gigina[1], Steven R. Talbot[3], Sergej Erschow[1], Dirk Wedekind[3], Robert Geffers[4], Norbert B. Ghyselinck[5], Heike Bähre[6], Jan C. Kamp[7, 8], Lavinia Neubert[2, 8], Melanie Ricke-Hoch[1], Johann Bauersachs[1], Christian Riehle[1, *]

[1] Department of Cardiology and Angiology, Hannover Medical School, Hannover, Germany
[2] Institute of Pathology, Hannover Medical School, Hannover, Germany
[3] Institute for Laboratory Animal Science, Hannover Medical School, Hannover, Germany
[4] Helmholtz Center for Infection Research, Research Group Genome Analytics, Braunschweig, Germany
[5] Institut de Génétique et de Biologie Moléculaire et Cellulaire (IGBMC), Département de Génétique Fonctionnelle et Cancer, Centre National de la Recherche Scientifique (CNRS UMR7104), Institut National de la Santé et de la Recherche Médicale (INSERM U1258), Université de Strasbourg, Illkirch, France
[6] Research Core Unit Metabolomics, Institute of Pharmacology, Hannover Medical School, Hannover, Germany
[7] Department of Respiratory Medicine, Hannover Medical School, Hannover, Germany
[8] German Center for Lung Research (DZL), Biomedical Research in Endstage and Obstructive Lung Disease Hannover (BREATH), Hannover, Germany

† These authors have contributed equally to this work and share first authorship.

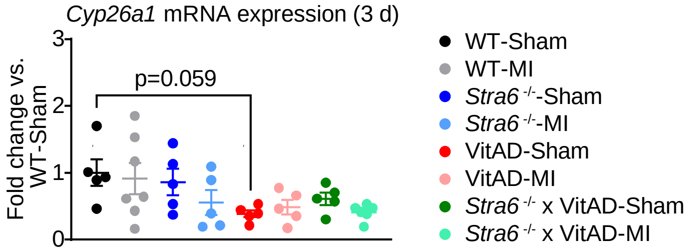

Supplementary Figure 1. *Cyp26a1 mRNA expression 3 days post-surgery*.
Cyp26a1 mRNA expression in the infarct border zone determined by RT-PCR analysis 3-days post-surgery normalized to *Gapdh* and presented as fold change vs. WT-Sham ($; n=5-7). Data are reported as mean values ± SEM. Two-way ANOVA was performed to analyze differences between Sham-operated groups ($ p<0.05 for VitAD) and MI-operated groups by *Stra6*$^{-/-}$ and VitAD.

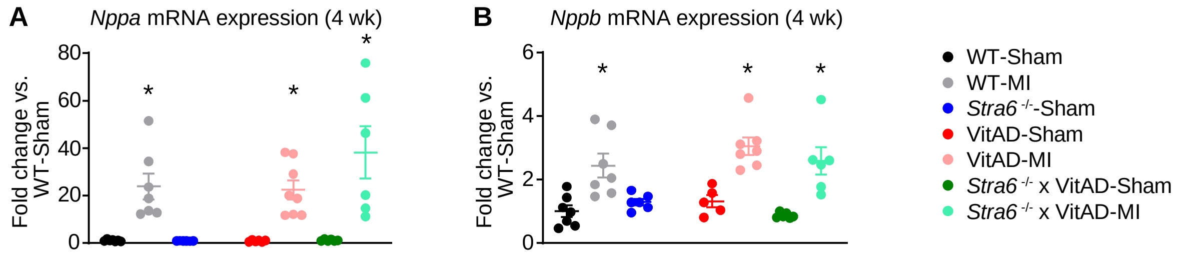

Supplementary Figure 2: *mRNA expression of heart failure markers 4 weeks post-surgery.* (A/B) mRNA expression of (A) *Nppa* and (B) *Nppb* (&) in the infarct border zone 4 weeks post-surgery each normalized to *Gapdh* and presented as fold change vs. WT-Sham (n=5-8). Data are reported as mean values ± SEM. * $p<0.05$ vs. Sham same *Stra6* expression and vitamin A availability. Two-way ANOVA was performed to analyze differences between Sham-operated groups by *Stra6*$^{-/-}$ and VitAD (& $p<0.05$ for the interaction between *Stra6*$^{-/-}$ and VitAD).

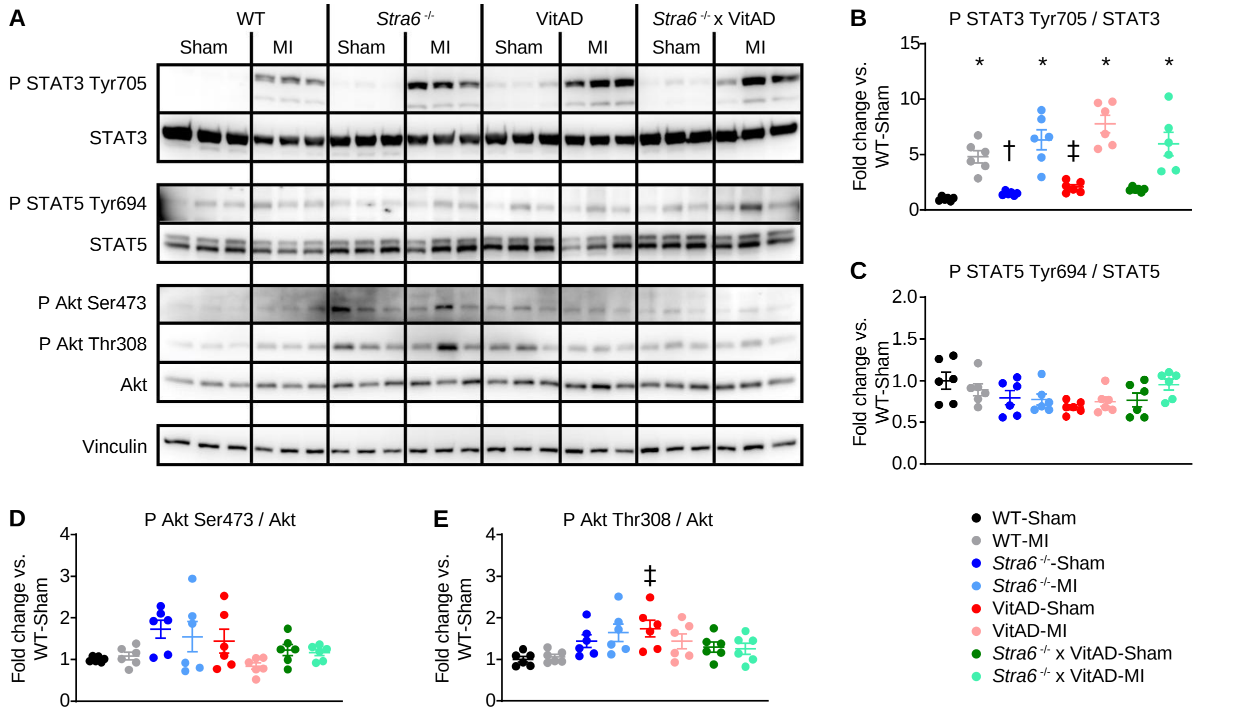

Supplementary Figure 3. *STAT3, but not STAT5/Akt signaling is activated following ischemic injury independent of Stra6 expression and vitamin A availability 3 days post-surgery.*
(A) Representative immunoblots in the infarct border zone and densitometric quantification of (B) P STAT3 Tyr705 normalized to STAT3 ($, &), (C) P STAT5 Tyr694 normalized to STAT5 ($, &&), (D) P Akt Ser473 normalized to Akt (&), and (E) P Akt Thr308 normalized to Akt (&, &&) presented as fold change vs. WT-Sham (n=6). Data are reported as mean values ± SEM. * $p<0.05$ vs. Sham same *Stra6* expression and vitamin A availability, † $p<0.05$ vs. *Stra6*$^{+/+}$ same surgery and vitamin A availability, ‡ $p<0.05$ vs. vitamin A sufficiency same surgery and *Stra6* expression. Two-way ANOVA was performed to analyze differences between Sham-operated groups by *Stra6*$^{-/-}$ and VitAD ($ $p<0.05$ for VitAD and & $p<0.05$ for the interaction between *Stra6*$^{-/-}$ and VitAD). Two-way ANOVA was performed to analyze differences between MI-operated groups by *Stra6*$^{-/-}$ and VitAD (&& $p<0.05$ for the interaction between *Stra6*$^{-/-}$ and VitAD).

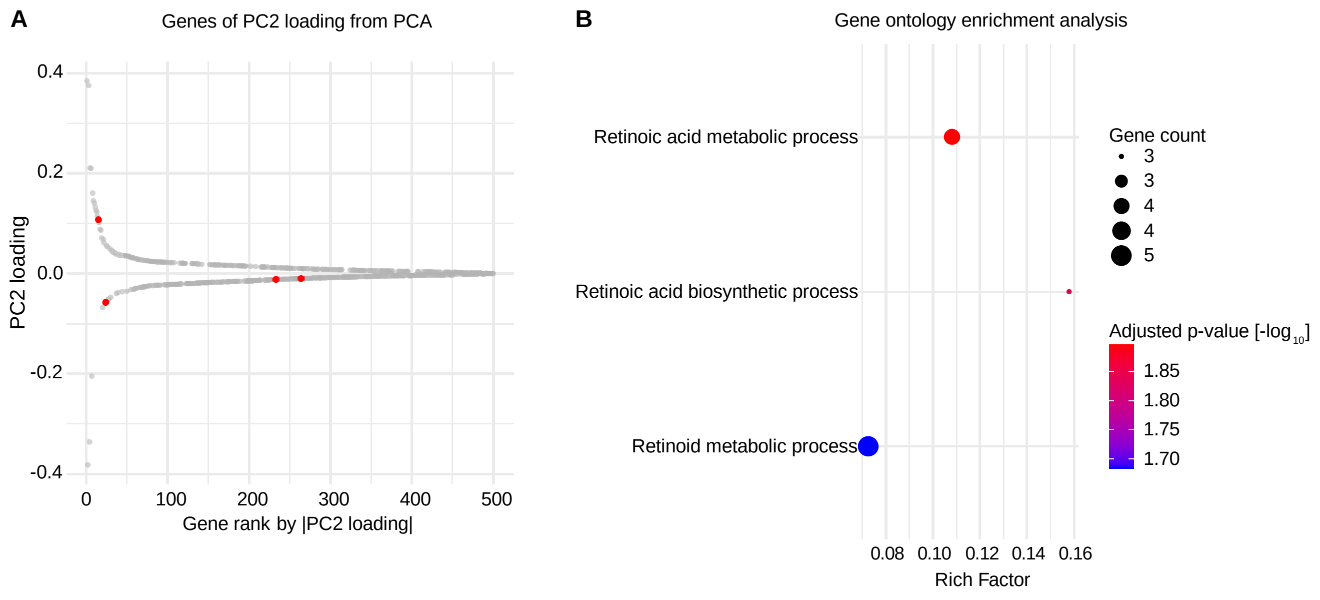

Supplementary Figure 4. *Retinoid signaling and metabolism genes contribute to principal component 2 of gene profiling.*
(A) Genes of principal component (PC) 2 loading from principal component analysis (PCA). Retinoid signaling and metabolism genes are highlighted with red. (B) Gene ontology enrichment analysis using the top 500 PC2-contributing genes, which identifies significantly enriched biological processes related to retinoid signaling/metabolism.

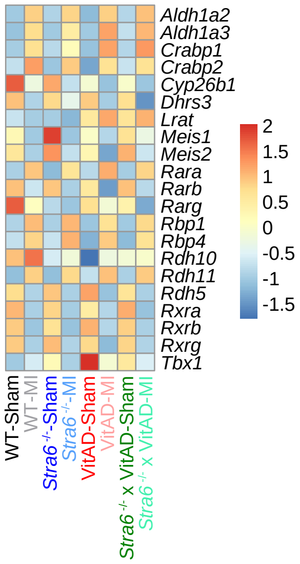

Supplementary Figure 5. *Expression of retinoid signaling and metabolism genes 3 days post-surgery.* Heatmap of RNA sequencing count data corresponding to canonical retinoid signaling and metabolism genes. Data were scaled by rows after applying the regularized log transformation function in DESeq2.

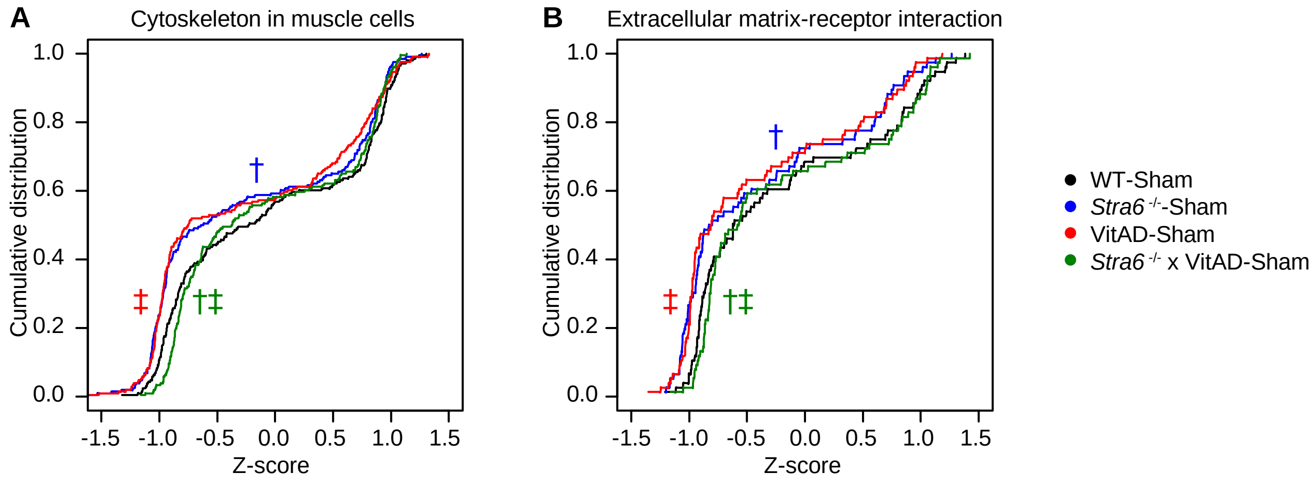

Supplementary Figure 6. *Expression of cytoskeleton and extracellular matrix (ECM)-receptor interaction genes in Sham-operated groups as determined by RNA sequencing.*

Cumulative distribution plots for Z-scores of (A) cytoskeleton in muscle cells and (B) extracellular matrix (ECM)-receptor interaction genes based on KEGG pathways. A shift to the right indicates an increased abundance of pathway genes. † $p<0.05$ vs. *Stra6*$^{+/+}$ and same vitamin A availability, ‡ $p<0.05$ vs. vitamin A sufficiency same *Stra6* expression (two-sided Kolmogorov-Smirnov test).

Supplementary Table 1: *Primers used for quantitative RT-PCR analysis.*

**Gene name *(Gene Symbol)*<br>Sequence of forward and<br>reverse primers (5' → 3')**
Cytochrome P450, family 26, subfamily a, polypeptide 1 *(Cyp26a1)*<br>ACCCACATGTCCTCCAGAAA<br>AGTCTTCAGAGCAACCCGAA
Glyceraldehyde-3-phosphate dehydrogenase *(Gapdh)*<br>CGTCCCGTAGACAAAATGGT<br>GAATTTGCCGTGAGTGGAGT
Lecithin-retinol acyltransferase *(Lrat)*<br>TCCCTCCTCCTGGAGAAACT<br>GCCAGGCCTGTGTAGACAAT
Natriuretic peptide type A *(Nppa)*<br>ATTGACAGGATTGGAGCCCAGAGT<br>TGACACACCACAAGGGCTTAGGAT
Natriuretic peptide type B *(Nppb)*<br>CTCAAGCTGCTTTGGGCACAAGAT<br>AGCCAGGAGGTCTTCCTACAACAA
Secreted frizzled-related protein 5 *(Sfrp5)*<br>GATGGCCTCATGGAACAGAT<br>CAGGACCAGCTTCTTGGTGT
Stimulated by retinoic acid gene 6 *(Stra6)*<br>GTCATCGCGGACTTGGAC<br>TTGATGCTGCAGTGAGGTTC
Wingless-type MMTV integration site family, member 4 *(Wnt4)*<br>GCGTAGCCTTCTCACAGTCC<br>ACGTCTTTACCTCGCAGGAG
Wingless-type MMTV integration site family, member 5B *(Wnt5b)*<br>TGGAGACAACGTGGAGTACG<br>GGCGACATCAGCCATCTTAT
Wingless-type MMTV integration site family, member 7A *(Wnt7a)*<br>GAGAAGCAAGGCCAGTACCA<br>TGGTCCAGCACGTCTTAGTG

Supplementary Table 2: *Antibodies used for immunoblotting.*

Antigen	Company	Catalog number
P Akt Ser473	Cell Signaling, Danvers, MA, USA	9271
P Akt Thr308	Cell Signaling, Danvers, MA, USA	9275
Akt	Cell Signaling, Danvers, MA, USA	9272
P STAT3 Tyr705	Cell Signaling, Danvers, MA, USA	9145
STAT3	Cell Signaling, Danvers, MA, USA	12640
P STAT5 Tyr694	Abcam, Cambridge, MA, USA, USA	ab32364
STAT5	Cell Signaling, Danvers, MA, USA	94205
Vinculin	Santa Cruz Biotechnology, Santa Cruz, CA, USA	7649
Anti-goat HRP-linked	Jackson ImmunoResearch, West Grove, PA, USA	305-035-045
Anti-rabbit HRP-linked	GE HealthCare, Chicago, IL, USA	NA934V

Supplementary Table 3: *Contractile function as assessed by transthoracic echocardiography.*

Time point	Group (n)	Heart rate [bpm]	LVEDA [mm^2]	LVESA [mm^2]	Ejection fraction [%]	FAC [%] #
**3 d.**	WT-Sham (7)	540 ± 15	24.1 ± 1.5	14.5 ± 1.2	59.4 ± 2.2	40.3 ± 1.9
	WT-MI (7)	560 ± 8	29.6 ± 1.6 *	24.5 ± 1.6 *	28.3 ± 4.0 *	17.5 ± 2.5 *
	Stra6$^{-/-}$-Sham (7)	545 ± 30	24.5 ± 1.8	15.6 ± 1.3	55.0 ± 1.8	36.5 ± 1.3
	Stra6$^{-/-}$-MI (6)	581 ± 36	29.5 ± 1.1 *	24.6 ± 1.2 *	24.7 ± 3.4 *	16.6 ± 2.4 *
	VitAD-Sham (6)	577 ± 34	24.3 ± 0.6	14.3 ± 0.7	60.9 ± 2.8	41.4 ± 2.1
	VitAD-MI (7)	586 ± 17	28.5 ± 1.4 *	23.3 ± 1.6 *	28.1 ± 4.3 *	18.6 ± 2.9 *
	Stra6$^{-/-}$ x VitAD-Sham (6)	553 ± 29	25.6 ± 2.2	16.3 ± 2.0	55.8 ± 2.8	37.1 ± 2.3
	Stra6$^{-/-}$ x VitAD-MI (6)	573 ± 28	33.5 ± 1.6 *	29.7 ± 1.9 *	20.6 ± 4.1 *	11.9 ± 2.0 *
**Time point**	**Group (n)**	**Heart rate [bpm]**	**LVEDA [mm^2]**	**LVESA [mm^2]**	**Ejection fraction [%]**	**FAC [%]**
**2 wk.**	WT-Sham (6)	563 ± 27	25.1 ± 1.3	15.8 ± 1.7	54.7 ± 5.9	37.3 ± 4.5
	WT-MI (9)	577 ± 28	34.2 ± 3.2 *	27.7 ± 3.5 *	33.8 ± 5.7 *	21.5 ± 3.5 *
	Stra6$^{-/-}$-Sham (6)	578 ± 26	23.3 ± 1.4	14.5 ± 1.9	55.7 ± 6.3	38.8 ± 5.7
	VitAD-Sham (6)	604 ± 15	25.0 ± 2.3	16.7 ± 2.3	51.6 ± 5.1	34.5 ± 3.8
	VitAD-MI (9)	588 ± 28	31.9 ± 2.6	25.3 ± 3.1	33.3 ± 5.7 *	22.8 ± 4.2
	Stra6$^{-/-}$ x VitAD-Sham (6)	606 ± 20	21.9 ± 1.5	13.4 ± 1.3	57.9 ± 3.3	39.2 ± 2.7
	Stra6$^{-/-}$ x VitAD-MI (7)	565 ± 19	38.5 ± 1.0 *	33.8 ± 0.9 *	18.0 ± 2.6 *	12.2 ± 1.5 *
**Time point**	**Group (n)**	**Heart rate [bpm] #**	**LVEDA [mm^2]**	**LVESA [mm^2]**	**Ejection fraction [%]**	**FAC [%]**
**4 wk.**	WT-Sham (7)	575 ± 28	23.4 ± 1.7	14.7 ± 1.5	54.5 ± 4.5	37.6 ± 4.1
	WT-MI (9)	581 ± 25	36.6 ± 3.3 *	29.4 ± 3.5 *	33.7 ± 4.6 *	21.5 ± 2.8 *
	Stra6$^{-/-}$-Sham (6)	510 ± 32	25.3 ± 1.1	15.7 ± 0.6	56.2 ± 3.1	37.5 ± 2.9
	VitAD-Sham (6)	620 ± 11	24.2 ± 1.6	14.7 ± 1.9	58.2 ± 5.6	40.0 ± 4.3
	VitAD-MI (9)	606 ± 13	35.6 ± 1.7 *	28.4 ± 2.0 *	32.5 ± 3.8 *	20.7 ± 2.6 *
	Stra6$^{-/-}$ x VitAD-Sham (6)	603 ± 13	23.3 ± 1.1	14.7 ± 1.2	55.2 ± 4.5	37.2 ± 4.0
	Stra6$^{-/-}$ x VitAD-MI (7)	585 ± 21	41.4 ± 2.3 *	34.3 ± 1.7 *	26.2 ± 1.9 *	16.9 ± 1.3 *

Bpm, beats per minute; FAC, fractional area change; LVEDA, left ventricular end-diastolic area; LVESA, left ventricular end-systolic area. Data are reported as mean values ± SEM. * $p<0.05$ vs. Sham same *Stra6* expression and vitamin A availability. Two-way ANOVA was performed to analyze differences between Sham-operated groups by *Stra6*$^{-/-}$ and VitAD (# $p<0.05$ for *Stra6*$^{-/-}$) and between MI-operated groups the 3-day time point (no significance detected).

Supplementary Table 4: *Morphometrics of mice.*

Time point	Group (n)	BW [g] #	HW [mg] &, $$	WLW [mg]	TL [mm] ##	HW/TL [mg/mm] &, $$	WLW/TL [mg/mm]
3 d.	WT-Sham (7)	26.9 ± 0.9	143.3 ± 4.9	140.7 ± 3.4	16.4 ± 0.1	8.75 ± 0.33	8.59 ± 0.19
	WT-MI (7)	25.1 ± 0.6	174.9 ± 3.6 *	156.4 ± 9.2	16.4 ± 0.1	10.65 ± 0.21 *	9.53 ± 0.54
	Stra6$^{-/-}$-Sham (6)	27.3 ± 0.8	126.2 ± 2.9 †	139.7 ± 8.3	16.5 ± 0.1	7.67 ± 0.14 †	8.48 ± 0.46
	Stra6$^{-/-}$-MI (6)	26.4 ± 0.9	157.5 ± 5.8 *	133.7 ± 4.5	16.0 ± 0.1 *	9.82 ± 0.36 *	8.34 ± 0.30
	VitAD-Sham (5)	25.0 ± 0.6	130.0 ± 4.0	132.6 ± 4.2	16.2 ± 0.1	8.03 ± 0.20	8.20 ± 0.26
	VitAD-MI (7)	25.0 ± 0.5	180.1 ± 8.9 *	164.3 ± 19.1	16.4 ± 0.1	11.03 ± 0.61 *	10.08 ± 1.26
	Stra6$^{-/-}$ x VitAD-Sham (6)	29.2 ± 1.4	148.8 ± 3.4 †, ‡	148.3 ± 3.2	16.4 ± 0.2	9.06 ± 0.21 †, ‡	9.03 ± 0.23
	Stra6$^{-/-}$ x VitAD-MI (6)	24.9 ± 1.0 *	188.8 ± 13.6 *	174.2 ± 21.5	16.2 ± 0.2	11.65 ± 0.77 *	10.77 ± 1.35
**Time point**	**Group (n)**	**BW [g] $**	**HW [mg] $**	**WLW [mg]**	**TL [mm] #**	**HW/TL [mg/mm]**	**WLW/TL [mg/mm]**
4 wk.	WT-Sham (6)	29.0 ± 0.7	147.0 ± 7.9	147.2 ± 2.7	17.3 ± 0.1	8.51 ± 0.45	8.52 ± 0.16
	WT-MI (9)	27.5 ± 0.8	159.4 ± 6.1	147.0 ± 10.8	16.6 ± 0.1 *	9.59 ± 0.34	8.85 ± 0.66
	Stra6$^{-/-}$-Sham (6)	29.4 ± 0.7	136.0 ± 6.0	140.0 ± 5.5	16.2 ± 0.1 †	8.40 ± 0.39	8.64 ± 0.35
	VitAD-Sham (6)	26.1 ± 0.4	128.3 ± 2.5	144.8 ± 4.9	16.8 ± 0.3	7.64 ± 0.26	8.64 ± 0.44
	VitAD-MI (9)	25.2 ± 0.8	170.9 ± 5.1 *	179.1 ± 28.8	16.6 ± 0.2	10.29 ± 0.29 *	10.77 ± 1.70
	Stra6$^{-/-}$ x VitAD-Sham (6)	27.0 ± 1.5	129.0 ± 6.0	138.3 ± 8.8	16.3 ± 0.2	7.89 ± 0.34	8.46 ± 0.49
	Stra6$^{-/-}$ x VitAD-MI (7)	25.7 ± 0.6	178.4 ± 5.1 *	167.9 ± 16.9	16.3 ± 0.2	10.95 ± 0.35 *	10.32 ± 1.07

BW, body weight; HW, heart weight; TL, tibia length; WLW, wet lung weight. Data are reported as mean values ± SEM. * $p<0.05$ vs. Sham same *Stra6* expression and vitamin A availability, † $p<0.05$ vs. *Stra6*$^{+/+}$ same surgery and vitamin A availability, ‡ $p<0.05$ vs. vitamin A sufficiency same surgery and *Stra6* expression. Two-way ANOVA was performed to analyze differences between Sham-operated groups by *Stra6*$^{-/-}$ and VitAD (# $p<0.05$ for *Stra6*$^{-/-}$, $ $p<0.05$ for VitAD, and & $p<0.05$ for the interaction between *Stra6*$^{-/-}$ and VitAD). Two-way ANOVA was performed to analyze differences between MI-operated groups by *Stra6*$^{-/-}$ and VitAD at the 3-day time point (## $p<0.05$ for *Stra6*$^{-/-}$ and $$ $p<0.05$ for VitAD).
